# Alcohol Withdrawal with Delirium Tremens

**DOI:** 10.21980/J8S35N

**Published:** 2023-07-31

**Authors:** Courtney Schwebach, Amrita Vempati

**Affiliations:** * Creighton University School of Medicine Phoenix Program, Valleyhealth Medical Center, Department of Emergency Medicine, Phoenix, AZ

## Abstract

**Audience:**

Emergency medicine (EM) residents (1^st^ year and 2^nd^ year levels), 4th year medical students and advanced practice providers

**Introduction:**

Alcohol use has played a major role in causing significant morbidity and mortality for patients. In 2016, it was the 7th leading risk factor for deaths and disability-adjusted life years globally.^1^ Among heavy alcohol users admitted for hospital management, the incidence of alcohol withdrawal syndrome is estimated to be 1.9 to 6.7%.^1^ Alcohol withdrawal (AW) in the ED has been associated with increased use of critical care resources, and frequent ED visits for alcohol-related presentations have been associated with mortality rates that are about 1–4% when withdrawal progresses to delirium tremens (DTs).^1^ Patients with alcohol withdrawal can present in many different ways to the ED including anxiety, tachycardia, delirium tremens (DTs), seizures and severe autonomic dysfunction leading to severe sickness and death.^2^ Therefore, it is extremely important for an EM physician to recognize the signs of AW in patients and to manage the critically ill patients. In addition, Clinical Institute Withdrawal Assessment (CIWA) of alcohol was developed to assess severity of alcohol withdrawal in 1989.^3^ EM physicians should utilize CIWA to help determine the severity of AW.

**Educational Objectives:**

By the end of the session, learner will be able to 1) discuss the causes of altered mental status, 2) utilize CIWA scoring system to quantify AW severity, 3) formulate appropriate treatment plan for AW by treating with benzodiazepine and escalating treatment appropriately, 4) treat electrolyte abnormalities by giving appropriate medications for hypokalemia and hypomagnesemia, and 5) discuss clinical progression and timing to AW.

**Educational Methods:**

This session was conducted using high-fidelity simulation, which was immediately followed by an in-depth debriefing session. The session was run during first year EM resident intern orientation, and it was run during two consecutive years. There was a total of 32 EM residents who participated. There was a total of 16 residents who actively managed the patient while the other 16 were observers. Each session had four learners and was run twice in two separate rooms. There was one simulation instructor running the session and one simulation technician who acted as a nurse.

**Research Methods:**

After the simulation and debriefing session was complete, an online survey was sent via surveymonkey.com to all the participants. The survey collected responses to the following questions: (1) the case was believable, (2) the case had right the amount of complexity (based on their Gestalt), (3) the case helped in improving medical knowledge and patient care, (4) the simulation environment gave me a real-life experience and, (5) the debriefing session after simulation helped improve my knowledge. The responses were collected using a Likert scale of 1 to 5 with 1 being “Strongly disagree” and 5 being “Strongly agree.”

**Results:**

There was a total of 15 respondents from both years. One hundred percent of them either agreed or strongly agreed that the case was beneficial in learning, in improving medical knowledge and in patient care. All of them found the post-session debrief to be very helpful. Two of them felt neutral about the case being realistic. The median response for questions 1, 3 and 5 is 5. The median response for questions 2 and 4 was 4. The range of responses for questions 1, 2, 3 and 5 was 4–5 while the range for question 4 was 3–5.

**Discussion:**

This high-fidelity simulation was a cost-effective and realistic way of educating learners on how to manage AW with DTs. Learners are forced to start with a broad differential for the patient who presents with AMS. As they recognize the cause of mental status, the patient quickly decompensates into developing severe agitation and autonomic dysfunction requiring learners to manage the patient and establish an airway. Learners found the case to be beneficial in learning the management of AW.

**Topics:**

Alcohol withdrawal, delirium tremens, agitation, altered mental status.

## USER GUIDE

[Table t2-jetem-8-3-s1]

**Table t2-jetem-8-3-s1:** 

List of Resources:	
Abstract	1
User Guide	3
Instructor Materials	5
Operator Materials	16
Debriefing and Evaluation Pearls	21
Simulation Assessment	27

**Learner Audience:**

Medical Students, Interns, Junior Residents, Advanced Practice Providers (PAs, NPs)

**Time Required for Implementation:**

**Instructor Preparation:** 20–30 minutes

**Time for case:** 15–20 minutes

**Time for debriefing:** 30–40 minutes

**Recommended Number of Learners per Instructor:**

3–4

**Topics:**

Alcohol withdrawal, delirium tremens, agitation, altered mental status.

**Objectives:**

By the end of the session, learner will be able to

1. Discuss the causes of altered mental status
2. Utilize CIWA scoring system to quantify AW severity
3. Formulate appropriate treatment plan for AW by treating with benzodiazepine and escalating treatment appropriately
4. Treat electrolyte abnormalities by giving appropriate medications for hypokalemia and hypomagnesemia
5. Discuss clinical progression and timing to AW

### Linked objectives and methods

Altered mental status (AMS) is one of the most common presentations to the emergency department (ED). The patient presents to the ED with AMS and the patient has tremors and severe agitation. Learners will have to consider various causes of AMS and elicit the regular alcohol use history (objective #1). After the history of alcohol use is obtained, learners will need to calculate CIWA score, which is very high in the simulated patient (objective #2). Learners will need to proceed forward by treating the patient with benzodiazepines and escalating the dosing when needed (objective #3). In addition, the patient also has hypokalemia and hypomagnesemia on the labs which will need to be addressed (objective #4). After the session is complete, during the debrief section, learners will need to discuss the clinical and time progression of AW (objective #5).

### Recommended pre-reading for instructor

- Yanta J, Swartzentruber G, Pizon A. Alcohol withdrawal syndrome: improving outcomes in the emergency department with aggressive management strategies. *Emerg Med Pract*. 2021 Mar 15;23(Suppl 3):1–41. PMID: 33729735.
- Farkas J. Alcohol withdrawal. Published July 21, 2022. Accessed August 26, 2022. EMCrit Project. At: https://emcrit.org/ibcc/etoh/

### Results and tips for successful implementation

This session was conducted with a total of 32 EM interns—a total of 16 interns managed the case while the rest were observers. One actor served as a nurse. Allowing the team to assign roles prior to starting the case helped run the case smoothly.

Depending on the level of the learners, prompting by the nurse may be required to notify them that the patient is very agitated and diaphoretic. Using the seizure function on the manikin may be helpful to simulate the tremulousness and/or agitation in the patient. If the learners do not administer benzodiazepines, the patient will continue to worsen and progress to seizure. Learners will need to establish an airway as the case progresses since the patient will be very altered. Novice learners may need guidance by a nurse consultant to administer magnesium if not done already.

After the simulation and debriefing session was completed, an online survey was sent via surveymonkey.com to all 32 participants. The responses were collected using a Likert scale of 1 to 5 with 1 being “Strongly disagree” and 5 being “Strongly agree.” The survey collected responses to the following questions:

1. The case was believable.
2. The case had a right amount of complexity (based on their Gestalt).
3. The case helped improve medical knowledge and patient care.
4. The simulation environment gave me a real-life experience.
5. The debriefing session after simulation helped improve my knowledge.

**Chart 1:**
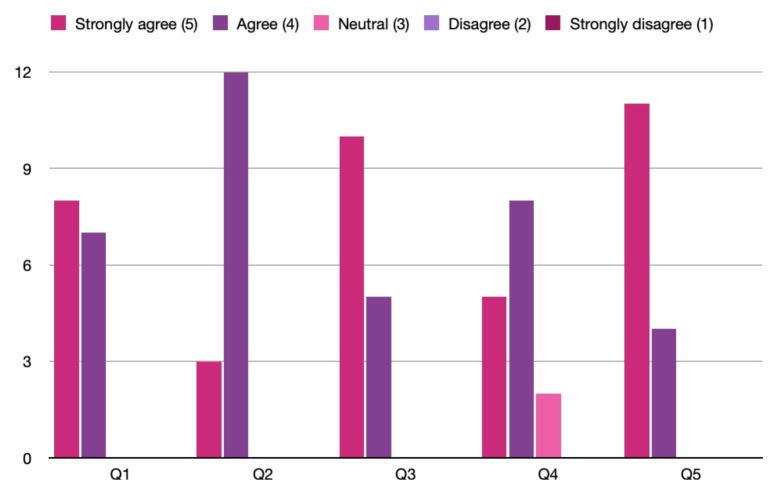
Survey Responses on Likert Scale

There were a total of 15 respondents from both years. One hundred percent of them either agreed or strongly agreed that the case was beneficial in learning, in improving medical knowledge, and in patient care. All of them found the post-session debrief to be very helpful. Two of them felt neutral about the case being realistic. The results are shown in Chart 1.[Table t3-jetem-8-3-s1]

**Table t3-jetem-8-3-s1:** 

Survey questions	Median	Range
Q1. The case was believable.	5	4–5
Q2. The case had a right amount of complexity (based on their Gestalt).	4	4–5
Q3. The case helped improvie medical knowledge and patient care.	5	4–5
Q4. The simulation environment gave me a real-life experience.	4	3–5
Q5. The debriefing session after simulation helped improve my knowledge.	5	4–5

The table above shows the median and range values based on allotting number values to each of the Likert scale responses as shown in the graph. The median response for questions 1, 3 and 5 is 5, which indicates that the majority of the respondents strongly agreed that the case was believable, that it helped improve patient care and knowledge, and that the debriefing section was helpful. The median response for questions 2 and 4 was 4, which indicates that the majority of the respondents agreed that the case had the right amount of complexity and that the simulation environment gave the learners real-life experience.

The range of responses for questions 1, 2, 3 and 5 was 4–5, which indicates that learners either agreed or strongly agreed that the case was believable, that it had the right amount of complexity, that it helped improve their medical knowledge and patient care, and that the debriefing session after simulation was helpful. The range for question 4 was 3–5, which shows that while the majority of the learners agreed or strongly agreed that the simulation gave them real-life experience, a few of them felt neutral about the case being realistic.

The results show that the case was helpful in improving learner’s medical knowledge and that the debrief session was helpful in learning about the case. Furthermore, the majority of the learners agreed that the case gave them real-life experience and that the case had the right amount of complexity.

Comments from the survey:

- “Great debriefing session! Provided a lot of good information when it comes to recognizing and treating alcohol withdrawal. Maybe cover dosing a little more for the phenobarb, other than that it was awesome!”
- “Very tough but great case!”
- “Overall, great case ”
- “Very realistic”

## Supplementary Information



## Figures and Tables

**Table t4-jetem-8-3-s1:** 

#1	Electrocardiogram (ECG)
#2	Complete blood count (CBC)
#3	Comprehensive metabolic panel (CMP)
#4	Lactate
#5	Magnesium level
#6	Coagulation panel
#7	Lipase
#8	Serum toxicologic screen
#9	Urinalysis
#10	Urine toxicologic screen
#11	Chest X-ray (CXR)
#12	Computerized tomography (CT) scan of the head

**Table t5-jetem-8-3-s1:** 

**Vitals:**	Heart Rate (HR) 161	Blood Pressure (BP) 191/100
	Respiratory Rate (RR) 25	Temperature (T) 37.4°C
	Oxygen Saturation (O_2_ Sat) 99% on room air	

**Figure f2-jetem-8-3-s1:**
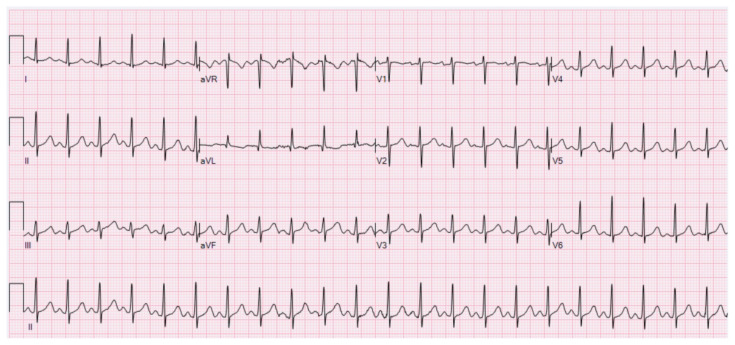


**Table t6-jetem-8-3-s1:** 

*Complete blood count (CBC)*	
White blood count (WBC)	9.6 **×** 1000/mm^3^
Hemoglobin (Hgb)	10.1 g/dL
Hematocrit (HCT)	30.3%
Mean Corpuscular Volume (MCV)	105 fL
Platelet (Plt)	77 **×** 1000/mm^3^

**Table:** 

*Comprehensive metabolic panel (CMP)*	
Sodium	129 mEq/L
Potassium	3.2 mEq/L
Chloride	82 mEq/L
CO2	17 mmol/L
Blood Urea Nitrogen (BUN)	40 mg/dL
Creatinine (Cr)	2.0 mg/dL
Glucose	77 mg/dL
Calcium	9.0 mg/dL
Total Protein	5.5 g/dL
Albumin	2.8 g/dL
Total bilirubin	1.5 mg/dL
Alkaline phosphate	250 units/L
Aspartate aminotransferase (AST)	48 units/L
Alanine aminotransferase (ALT)	96 units/L

**Table t8-jetem-8-3-s1:** 

*Lactate*	1.2 mmol/L

**Table t9-jetem-8-3-s1:** 

*Magnesium*	1.5 mEq/L

**Table t10-jetem-8-3-s1:** 

*Coagulation Panel*	
International normalized ratio	1.3
Prothrombin time	14 seconds
Activated partial thromboplastin time	32 seconds

**Table t11-jetem-8-3-s1:** 

*Lipase*	67 units/L

**Table t12-jetem-8-3-s1:** 

*Serum toxicologic drug screen*	
EtOH	Negative
Salicylate	Negative
Acetaminophen	Negative

**Table t13-jetem-8-3-s1:** 

*Urinalysis*	
b-HCG	Negative
Color	Yellow
Appearance	Clear
Specific gravity	1.500
Leukocyte esterase	Negative
Nitrites	Negative
Blood	None
Protein	2+
Ketones	2+
Glucose	Negative
Bilirubin	Negative
White blood cells	0–5 WBCs/HPF
Red blood cells	0–5 RBCs/HPF
Squamous epithelial cells	0–5 cells/HPF

**Table t14-jetem-8-3-s1:** 

*Toxicology Screen*	
Amphetamines	Negative
Barbiturates	Negative
Benzodiazepines	Negative
Cocaine	Negative
Marijuana	Negative
Methadone	Negative
Methamphetamine	Positive
Opiates	Negative
Phencyclidine	Negative
Tricyclic antidepressants	Negative

**Figure f3-jetem-8-3-s1:**
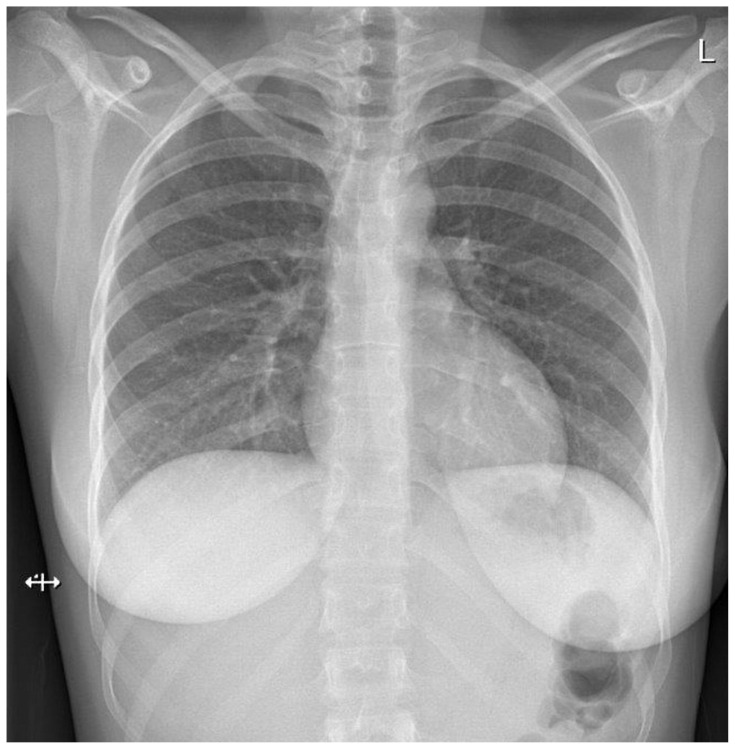


**Figure f4-jetem-8-3-s1:**
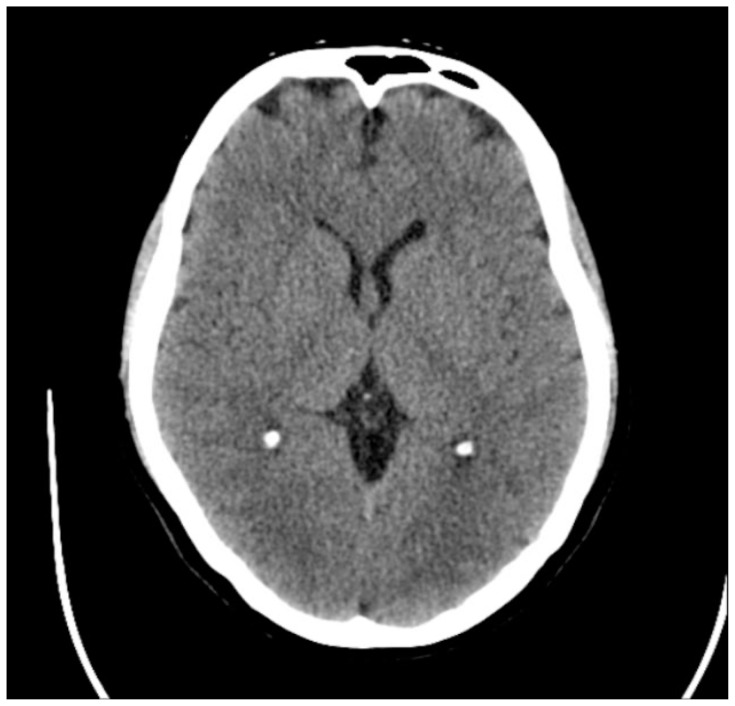


**Table t15-jetem-8-3-s1:** SIMULATION EVENTS TABLE:

Minute (state)	Participant action/trigger	Patient status (simulator response) & operator prompts	Monitor display (vital signs)
**State 1** 0:00 – 2:00 (Baseline)	Team assesses vital signs, obtains history, performs a complete physical exam including assessing neurological status. Obtain history from patient’s husband. Discuss with husband the diagnosis of alcohol withdrawal with delirium tremens, and ask if she has ever had this in the past.	Patient is agitated, yelling, “get the ants off of me.” Simulator: seizure activity is set to moderate. Nurse will bring husband inside when asked. Husband asks, “what is going on?!” If asked additional questions about drinking, husband states, “You know I have been finding a lot of empty vodka bottles hidden around the house, but she mentioned wanting to quit drinking completely with this new job.” When asked, point of care glucose 88 and provide ECG. Nursing can’t place IV because secondary to agitation.	T 37.4° C HR 161 BP 191/100 RR 25 O2 99%
**State 2** 2:00 – 5:00	Discuss the differential for altered mental status and/or agitation. Team will need to calculate CIWA. Order laboratory studies, ECG and imaging. Interpret ECG.	CIWA assessment: Nausea/vomiting: Constant nausea and frequent dry heaving (Score = 7). Tremor: Tremor with any movement (score = 7). Sweats: beads of sweat on forehead (score = 4). Agitation: moderately fidgety and moderately restless (score = 5). Tactile disturbances: mild itching, pin and needles, burning or numbness (score = 2). Auditory disturbances: mild harshness or ability to frighten (score = 2). Visual disturbances: mild sensitivity (score = 2). Headache, fullness in head: mild headache (score = 2). Orientation: disoriented for date by no more than 2 calendar days (score = 2). Let learners come up with the CIWA which is 33; if they don’t calculate CIWA, nurse will ask, “What is the CIWA?”	T 37.4° C HR 160 BP 195/110 RR 27 O2 99%
**State 3** 5:00 – 8:00	Administer intramuscular benzodiazepines. Lorazepam (4mg based on CIWA protocol which can be given intramuscularly (IM)) or midazolam (2–4mg IM)	No change in patient status. Nursing cue “I can’t place an IV since she is very agitated, what should I do?” Lorazepam takes a few minutes longer to work than midazolam when given IM. Learners will need to be specific in dosing of the medications. If benzodiazepines are not given, patient will continue to be agitated and IV cannot be placed. If haloperidol is given, it will not work. Phenobarbital cannot be given until IV and monitor are placed. Learners may need cue to avoid giving phenobarbital IM.	T 37.4° C HR 155 BP 186/92 RR 22 O2 99%
**State 4** 8:00–10:00	Evaluate the persistent agitation in the patient and escalate benzodiazepine dosing. Participants agree on treatment plan and timing for benzodiazepine, ideally q15 minutes for lorazepam (4mg based on CIWA protocol IV or IM), or q5 minutes for diazepam (10mg) or q5 min for midazolam (2–4mg IV or IM) with reevaluation and doubling of the dose each time. Interpret labs and administer electrolye replacement and IVF. This includes: thiamine 100 mg IV for supplementation, and 500 mg. magnesium replacement should start at 1g given slowly, and potassium chloride as IV boluses at 10mEq every hour. IVF should be administered and should contain dextrose (D5-LR or D5NS).	Patient agitated, shaking and tremulous, continues to be confused and complains of ants. Patient calms down enough for IV to be placed; however, the effect is more delayed if lorazepam and diazepam are given IM. Nursing asks, “How often do you want me to give the medications, and is there any other treatment you want to give?” Learners will need to be specific about electrolyte replacement and dosage. If learners give phenobarbital 10–15mg/kg dose, proceed to state 7. If learners give phenobarbital 130 mg or 260 mg, then proceed to the next state 6.	T 37.4° C HR 150 BP 180/90 RR 20 O2 99%
**State 5** 10:00 – 13:00	Assess the mental status and deep tendon reflexes. Readminister benzodiazepine. Consider medication escalation to phenobarbital with loading dose of 10–15 mg/kg as a bolus. It can also be given in 130mg or 260mg boluses which can be repeated every 30 minutes as needed and titrated to symptom relief with maxiumum dose of 1040 mg in 24 hr.	Patient continues to remain tremulous, diaphoretic, and has tongue fasiculations. Nurse says, “ She’s still very agitated. What else can I give her?”	T 37.4° C HR 150 BP 185/98 RR 24 O2 99%
**State 6** 13:00 – 17:00	Assess patient’s airway and proceed to endotracheal intubation.	Patient is obtunded and not answering any questions or following commands. Learners will need to be establish airway while being specific about the equipment and medication dosages. Consider adding a nurse or husband prompt about the patient’s becoming less responsive, or adding something that would indicate an inability to protect her airway as the prompt for intubation.	T 37.4° C HR 140 BP160/95 RR 18 O2 88%
**State 7** 17:00 – 20:00	Provide patient with appropriate sedation and pain control. Propofol is the best sedation medication to use while intubated, and fentanyl for pain control. Discuss the case with the intensivist. Admit to the medical ICU.	Patient is intubated and sedated. Intensivist will agree to admit the medical ICU.	T 37.4° C HR 106 BP 149/82 RR vent O2 99%

**Table t16-jetem-8-3-s1:** 

**Critical Actions:** 1. Obtain point of care (POC) glucose 2. Ask husband for collateral history of alcohol use 3. Identify alcohol withdrawal with delirium tremens 8. Initiate treatment with benzodiazepine and escalate to higher dosing of medication 9. Assess deep tendon reflexes for additional benzodiazepine dosing 10. Initiate phenobarbital treatment when alcohol withdrawal is refractory to benzodiazepine administration 11. Recognize worsening mental status and establish airway Admit patient to ICU	0:00

**Table t17-jetem-8-3-s1:** 

	Milestone	Did not achieve level 1	Level 1	Level 2	Level 3
1	**Emergency Stabilization (PC1)**	□ Did not achieve Level 1	□ Recognizes abnormal vital signs	□ Recognizes an unstable patient, requiring intervention Performs primary assessment Discerns data to formulate a diagnostic impression/plan	□ Manages and prioritizes critical actions in a critically ill patient Reassesses after implementing a stabilizing intervention
2	**Performance of focused history and physical (PC2)**	□ Did not achieve Level 1	□ Performs a reliable, comprehensive history and physical exam	□ Performs and communicates a focused history and physical exam based on chief complaint and urgent issues	□ Prioritizes essential components of history and physical exam given dynamic circumstances
3	**Diagnostic studies (PC3)**	□ Did not achieve Level 1	□ Determines the necessity of diagnostic studies	□ Orders appropriate diagnostic studies. Performs appropriate bedside diagnostic studies/procedures	□ Prioritizes essential testing Interprets results of diagnostic studies Reviews risks, benefits, contraindications, and alternatives to a diagnostic study or procedure
4	**Diagnosis (PC4)**	□ Did not achieve Level 1	□ Considers a list of potential diagnoses	□ Considers an appropriate list of potential diagnosis May or may not make correct diagnosis	□ Makes the appropriate diagnosis Considers other potential diagnoses, avoiding premature closure
5	**Pharmacotherapy (PC5)**	□ Did not achieve Level 1	□ Asks patient for drug allergies	□ Selects an medication for therapeutic intervention, consider potential adverse effects	□ Selects the most appropriate medication and understands mechanism of action, effect, and potential side effects Considers and recognizes drug-drug interactions
6	**Observation and reassessment (PC6)**	□ Did not achieve Level 1	□ Reevaluates patient at least one time during case	□ Reevaluates patient after most therapeutic interventions	□ Consistently evaluates the effectiveness of therapies at appropriate intervals
7	**Disposition (PC7)**	□ Did not achieve Level 1	□ Appropriately selects whether to admit or discharge the patient	□ Appropriately selects whether to admit or discharge Involves the expertise of some of the appropriate specialists	□ Educates the patient appropriately about their disposition Assigns patient to an appropriate level of care (ICU/Tele/Floor) Involves expertise of all appropriate specialists
9	**General Approach to Procedures (PC9)**	□ Did not achieve Level 1	□ Identifies pertinent anatomy and physiology for a procedure Uses appropriate Universal Precautions	□ Obtains informed consent Knows indications, contraindications, anatomic landmarks, equipment, anesthetic and procedural technique, and potential complications for common ED procedures	□ Determines a back-up strategy if initial attempts are unsuccessful Correctly interprets results of diagnostic procedure
20	**Professional Values (PROF1)**	□ Did not achieve Level 1	□ Demonstrates caring, honest behavior	□ Exhibits compassion, respect, sensitivity and responsiveness	□ Develops alternative care plans when patients’ personal beliefs and decisions preclude standard care
22	**Patient centered communication (ICS1)**	□ Did not achieve level 1	□ Establishes rapport and demonstrates empathy to patient (and family) Listens effectively	□ Elicits patient’s reason for seeking health care	□ Manages patient expectations in a manner that minimizes potential for stress, conflict, and misunderstanding. Effectively communicates with vulnerable populations, (at risk patients and families)
23	**Team management (ICS2)**	□ Did not achieve level 1	□ Recognizes other members of the patient care team during case (nurse, techs)	□ Communicates pertinent information to other healthcare colleagues	□ Communicates a clear, succinct, and appropriate handoff with specialists and other colleagues Communicates effectively with ancillary staff

**Table 1:** Agitated, febrile, tachycardia, altered: Tips for differentiating alcohol withdrawal patient from other pathology with similarly presenting symptoms^1^,^4^

Differential Diagnosis	History	Exam	Diagnostics
**Infectious** (Meningitis, encephalitis, pyelonephritis, pneumonia, appendicitis, diverticulitis, etc.)	**Infectious symptoms based on system** Pulmonary: cough, shortness of breath Genital Urinary (GU): dysuria, nausea, vaginal discharge vomiting, flank pain, suprapubic abdominal pain GastrointestinaI (GI): abdominal pain, nausea, vomiting, diarrhea Central Nervous System (CNS): neck rigidity, numbness, headache Skin: redness, warmth, swelling tenderness (CMT), adnexal tenderness	**Vitals:** hypotension **Exam findings based on system:** Pulmonary: hypoxia, crackles, decreased breath sounds GU: suprapubic tenderness, Costovertebral angle (CVA) tenderness, vaginal discharge, cervical motion GI: abdominal tenderness, jaundice CNS: neck rigidity, AMS Skin: blisters, bullae, streaking, erythema	**Labs:** leukocytosis on CBC, elevated lactic acid, elevated procalcitonin, UA with positive nitrates, leukocyte esterase, pyuria, bacturia + blood cultures **Procedures:** lumber puncture (LP) showing elevated WBC with neutrophil predominance, bacteria, low glucose for bacterial meningitis, or lymphocytic pleocytosis, elevated protein in enecphalitis **Imaging:** Chest xray showing consolidation, CT abdomen showing appendicitis, diverticulitis, pelvic ultrasound showing tuboovarian abscess
**Endocrine** (Hypoglycemia, diabetic ketoacidosis, hyperosmolar hyperglycemic state [HHS], thyrotoxicosis)	**Hypoglycemia:** unintentional/intentional ingestion of hypoglycemic agent such as insulin, poor PO intake, vomiting, in known diabetic, patient with alcohol abuse, underlying liver disease **Diabetic Ketoacidosis (DKA)**: Known diabetic with new infection, pregnancy, myocardial infarction (MI), cerebrovascular accident (CVA), non-compliant with insulin triggering DKA. Weight loss, polydipsia, polyuria in unknown diabetic **HHS**: Elderly patient with poor access to hydration either due to mobility, environmental conditions etc, with dehydration **Thyrotoxicosis/Thyroid Strom:** Gradually worsening palpitations, weight loss, fatigue, generalized weakness, ingestion of levothyroxine	**Hypoglycemia:** altered mental status **DKA:** ill-appearing, poor skin turgor, dry mucous membranes, fruity scent to breath, tachypneic, Kussmaul breathing pattern **HHS:** dry mucus membranes, poor skin turgor, altered mental status, hypotension due to dehydration **Thyrotoxicosis/Thyroid Storm:** proptosis of eyes, lid lag, goiter, tremulous, hypertension, tachycardia, febrile	**Labs:** point-of-care glucose showing hypo- or hyperglycemia, elevated beta-hydroxybutyrate, metabolic acidosis anion gap, ketonuria, decreased thyroid stimulating hormone (TSH) in thyrotoxicosis
**Trauma** (Intracranial hemorrhage)	**History of traumatic incident:** fall, moving vehicle collision (MVC), assault **Past medical history:** bleeding disorders (hemophilia), connective tissue disorders, previous intracranial hemorrhage, family history of brain anyuresum **Medications:** blood thinners	**Vitals:** bradycardia, hypertension, bradynpea concerning for herniation **Exam:** scalp hematoma, hemotympanum, cerebrospinal fluid (CSF) rhinorrhea, Battle’s sign, Racoon eyes, deviated eyes, pupil abnormalities including blown pupil, focal neuro deficits, posturing, decreased Glasgow coma scale (GCS)	**Head CT:** demonstrating subdural hematoma, epidural hematoma, subarachnoid hemorrhage or intraparenchymal hemorrhage
**Toxicology** (Sympathomimetic intoxication, neuroleptic muscular syndrome (NMS), serotonin syndrome (SS), anti-cholinergic syndrome)	**Sympathomimetic intoxication:** ingestion of sympathomimetic agents such as methamphetamine, cocaine, MDMA **NMS:** symptoms after recently initiated or adjusted dopamine-receptor antagonist medications such as neuroleptic agents (haloperidol) or discontinuation of dopaminergic medications **SS:** rapid onset of symptoms after ingestion of serotonergic drugs such as selective serotonin reuptake inhibitors (SSRIs), monoamine oxidase inhibitors (MAOIs) **Anti-cholinergic syndrome:** ingestion of anti-cholinergic agent such as antihistamines, jimson weed, either due to intentional ingestion in suicide attempt or unintentional ingestion by young child, blurry vision, inability to urinate	**Sympathomimetic intoxication:** diaphoretic, hallucinations, tachypneic **NMS:** severe muscle rigidity commonly described as lead-pipe rigidity, labile blood pressure, diaphoresis, tremor **SS:** ocular clonus, clonus, hyperreflexia, diaphoresis **Anti-cholinergic syndrome:** mydriasis, signs of dehydration (dry mucous membranes), hallucinations, seizures, flushing, suprapubic distension from urinary retention, dry skin from anhidrosis, mydriasis	There is no lab or imaging study that can give you the diagnosis because it is a clinical diagnosis

## INSTRUCTOR MATERIALS

**Case Title:** Alcohol Withdrawal with Delirium Tremens

**Case Description & Diagnosis (short synopsis):** A 49-year-old female brought in by husband for “not acting right.” The history is given by the patient’s husband only since the patient is very altered. The husband reports that the patient has been having nausea and vomiting for about a day and a half prior to arrival. After further questioning, the husband reports that the patient has alcohol use history which includes more than 6-pack of beer every day for over 10 years.

In the ED, patient will be alert and oriented to self only with shakiness of upper extremities, visual hallucinations, and diaphoresis. She will also be very tachycardic and hypertensive. As the case progresses, the patient will continue to complain of visual hallucinations and be very tachycardic. Despite administration of benzodiazepines, she will continue to be tremulous and diaphoretic. After phenobarbital is administered, patient will begin vomiting, prompting intubation and admission to the intensive care unit (ICU).

**Equipment or Props Needed:**

- High-fidelity simulation manikin which has seizure capability
- Moulage: 3 plastic ants – 1 on left arm, right leg, and abdomen
- Airway supplies:
  ○ Nasal cannula
  ○ Non-rebreather (NRB) mask
  ○ Laryngoscope and blades
  ○ Endotracheal tube and stylet
  ○ Bag-valve mask
- IV supplies:
  ○ 2 18g angiocatheters
  ○ IV tubing
  ○ Normal saline
  ○ Lactated Ringer’s
- Medications:
  ○ Etomidate
  ○ Succinylcholine
  ○ Epinephrine
  ○ Sodium bicarbonate
  ○ Magnesium sulfate
  ○ Potassium Chloride
  ○ Diazepam
  ○ Midazolam
  ○ Lorazepam
  ○ Phenobarbital
  ○ Ondansetron
  ○ Haloperidol
  ○ Droperidol
- Cardiac monitor
- Defibrillator
- Gloves

**Confederates needed:**

Two actors are needed: one to play a nurse and the other to play husband

**Stimulus Inventory:** [Table t4-jetem-8-3-s1]

**Background and brief information:** A 49-year-old female brought in by husband for altered mental status.

**Initial presentation:** Patient is a 49-year-old female with a past medical history of alcohol abuse and is brought in by her husband for altered mental status.

Husband reports: He states that she had been nauseous and vomiting about a day and a half ago. He notes she was very sweaty but did not have a fever. She had told him at that time she thought she had a stomach bug. However, over the past day, the husband states that she has not been acting herself and has seemed more confused. She was also complaining of a headache. He notes that they have eaten the same foods, and he has not gotten sick. He reports that the patient seemed to be picking at her skin prior to the patient’s becoming more confused. Patient currently complaining of nausea, but no abdominal pain or vomiting. In the emergency department (ED), the patient is alert and oriented x 1.

**How the scene unfolds:** Upon initial presentation to the ED, learners will determine that the patient is not able to provide any history, and they will need to get the full history from the husband. After history and physical examination, the patient will be found to be agitated, tremulous, and hallucinating. Learners will need to give an appropriate dosing of medication (benzodiazepines) to help the patient with her vital signs and her symptoms. Depending on the type of benzodiazepines given, the duration of action will change. They will need to continue to escalate the dosing of the benzodiazepines and look for the effectiveness of the medication. They will also need to adjust the dosing of benzodiazepine by assessing the patient’s deep tendon reflexes, which will become decreased due to the sedating effects on the central nervous system. During this time, the ECG and laboratory results will be provided. Learners will need to address the electrolyte abnormalities in the laboratory results. Despite multiple administrations of the benzodiazepines, the patient will continue to be agitated. Learners will need to administer appropriate weight-based dosage of phenobarbital. After phenobarbital is administered, the patient will be altered and will require airway establishment by the learners. After airway management, the intensivist will need to be called for admission to the intensive care unit (ICU).

**Critical actions:**

1. Obtain point of care (POC) glucose
2. Ask husband for collateral history of alcohol use
3. Identify alcohol withdrawal with delirium tremens
4. Initiate treatment with benzodiazepine and escalate to higher dosing of medication
5. Assess deep tendon reflexes for additional benzodiazepine dosing
6. Initiate phenobarbital treatment when alcohol withdrawal is refractory to benzodiazepine administration
7. Recognize worsening mental status and establish airway
  Admit patient to ICU

**Case Title:** Alcohol Withdrawal with Delirium Tremens

**Chief Complaint:** “not acting right”[Table t5-jetem-8-3-s1]

**General Appearance:** slightly agitated, tremulous, diaphoretic, picking at things, protecting airway, alert and oriented to self

**Primary Survey:**

- **Airway:** “get these ants off of me”
- **Breathing:** Clear to auscultation bilaterally. No rales or rhonchi
- **Circulation:** Tachycardic with bilateral radial and dorsalis pedis pulses present

**History:**

- **History of present illness:** Patient is a 49-year-old female with a past medical history of alcohol abuse brought in by husband for “not acting right.”
  Husband reports: He states that she had been nauseous and began vomiting about a day and a half ago. He notes she was very sweaty but did not have a fever. She had told him at that time she thought she had a stomach bug. However, over the past day, the husband states that she has not been acting herself and has seemed more confused. She was also complaining of a headache. He notes that they have eaten the same foods, and he has not gotten sick. He also remembers that the patient seemed to be picking at skin prior to the patient becoming more confused. Patient currently complaining of nausea, but no abdominal pain or vomiting.
  In the emergency department (ED), the patient is alert and oriented x 1.
- **Past medical history:** Hypertension, hyperlipidemia
- **Past surgical history:** Cholecystectomy
- **Patient’s medications:** Lisinopril, atorvastatin
- **Allergies:** none
- **Social history:** The husband reports that the patient has alcohol use history which includes more than 6-pack of beer every day for over 10 years. Non-smoker. They used to party a lot when they were younger and used marijuana and cocaine but haven’t in years. She got a new job recently and was very excited about starting it.
- **Family history:** Unknown

**Secondary Survey/Physical Examination:**

- **General appearance:** agitated, tremulous in upper extremities, diaphoretic, picking at things, protecting airway, alert and oriented to self
- **HEENT:**
  ○ **Head:** Within normal limits
  ○ **Eyes:** Pupils are 3mm, round and reflective to light
  ○ **Ears:** Within normal limits
  ○ **Nose:** Within normal limits
  ○ **Throat:** dry mucous membranes
- **Neck:** No thyromegaly
- **Heart:** S1 and S2 normal but tachycardic. No murmurs or gallops
- **Lungs:** clear to auscultation bilaterally. No rales or rhonchi
- **Abdominal/GI:** Soft, non-tender, nondistended. No scars present. Hepatomegaly present. No rigidity, guarding or rebound tenderness
- **Genitourinary:** Within normal limits
- **Rectal:** Within normal limits
- **Extremities:** Within normal limits
- **Back:** Within normal limits
- **Neuro:** Alert and oriented to self only. Follows some commands intermittently. No cranial nerve deficits
- **Skin:** Diaphoretic, facial and palmar flushing
- **Lymph:** Within normal limits
- **Psych:** Agitated and reaching out to grab things. Keeps stating “get these ants off of me.”

*ECG*

Image source: Ewingdo, CC BY-SA 4.0 <https://creativecommons.org/licenses/by-sa/via Wikimedia Commons. Retrieved on August 11, 2022 from 4.0>, via Wikimedia Commons[Fig f2-jetem-8-3-s1][Table t6-jetem-8-3-s1][Table t8-jetem-8-3-s1][Table t9-jetem-8-3-s1][Table t10-jetem-8-3-s1][Table t11-jetem-8-3-s1][Table t12-jetem-8-3-s1][Table t13-jetem-8-3-s1][Table t14-jetem-8-3-s1]

*Chest X-Ray - Normal*

Image source: Bickle I. Normal chest radiograph - female. Case study, Radiopaedia.org. Retrieved on August 12, 2022 from https://doi.org/10.53347/rID-46418[Fig f3-jetem-8-3-s1]

*CT Head - Normal*

Image source: Cuete D. Normal CT brain. Case study, Radiopaedia.org. Retrieved on August 12, 2022 from https://doi.org/10.53347/rID-23768[Fig f4-jetem-8-3-s1]

## OPERATOR MATERIALS

[Table t15-jetem-8-3-s1]

**Diagnosis:**

Alcohol withdrawal with delirium tremens

**Disposition:**

Medical ICU

## DEBRIEFING AND EVALUATION PEARLS

### Alcohol Withdrawal with Delirium Tremens

**Pearls:** The differential for altered mental status and agitation may include:

- Trauma
- Infection- sepsis secondary to CNS, pulmonary, urinary, etc
- Endocrine etiology - thyrotoxicosis, hypoglycemia, diabetic ketoacidosis
- Toxic ingestion - cocaine, methamphetamines
- Withdrawal – benzodiazepine or alcohol
- Serotonin syndrome
- Neuroleptic malignant syndrome.

Patients presenting to the ED with alcohol withdrawal often can have their symptoms attributed to other causes.^1^ In addition, other underlying medication conditions can precipitate alcohol withdrawal. Due to this confounding clinical presentation, it is incredibly important for clinicians to maintain a high suspicion for alcohol withdrawal. This is especially true because the diagnosis is a clinical diagnosis and not based on one laboratory test or imaging result.^4^ Additionally, it is incredibly important to continue to evaluate for other causes of symptoms. While it is difficult to discern between these diagnoses initially, which is why it is important to evaluate for all of them, there are some clues discussed in Table 1 that can help the learner.

Learners need to understand that alcohol withdrawal is a disease spectrum presenting as anxiety to delirium tremens, the most extreme presentation with a mortality rate of 2–3%, as our patient did.^4^ Here are the stages of alcohol withdrawal and their time of onset:

- < 6 hours – uncomplicated withdrawal is marked by anxiety, headache, nausea, vomiting, insomnia, mild tremors and mild to moderate autonomic instability.
- 8–12 hours – alcohol hallucinosis is marked by auditory, visual, tactile, gustatory and olfactory hallucinations with a possibility of paranoia and delusions.
- 12–24 hours – alcohol withdrawal seizures with generalized tonic-clonic seizures that occur in clusters with up to 3% progressing to status epilepticus.
- 24–72 hours – alcohol withdrawal delirium which is marked with confusion and alteration in consciousness, severe autonomic changes and persistent hallucinations.^1^

A tool learners can use to aid their diagnosis is the clinical institute withdrawal assessment for alcohol (CIWA). This scoring system is helpful in risk-stratifying patients initially into treatment subgroups (oral benzodiazepines vs IV benzodiazepines) and likely disposition based on presenting symptoms and exam findings.^3^,^4^ In order to use the tool, a clinician ranks a patient on 10 separate questions/categories on a scale from 0 to 7 based on severity.^3^ These 10 categories include: nausea and vomiting, tremors, paroxysmal sweats, anxiety, agitation, tactile, auditory and visual disturbances, headache and orientation.^3^ Of the 10 categories, three rely on the clinician’s observation of the patient (tremors, paroxysmal sweats and agitation), while the rest of the questions rely on the patient’s answer. This introduces room for error due to the subjectivity of the scale based on a patient’s communicative ability (language, mental status, other comorbidities such as previous stroke with speech difficulties).

In addition, it is important for learners to recognize the limitations of CIWA, including its study population on healthy outpatient and inpatient psychiatric populations rather than emergency department or inpatient populations.^4^ Severe alcohol withdrawal should be managed aggressively with escalating doses of IV benzodiazepines with consideration of phenobarbital for benzo-resistant alcohol withdrawal early in treatment.^2^,^4^,^5^

The medications that can be given include:

1. Diazepam – It takes about 1–5 minutes for onset and initial dose can be 10–20mg given either IV or PO.
2. Lorazepam – Used more often than any other medications. The onset can be about 5–20 min if given IV and initial dose can be 2–4 mg IV or PO.
3. Midazolam – Very quick in onset with the timings only 2–5 min IV or IM and initial dosing is 2–4 mg IM/IV.
4. Chlordiazepoxide – Used more in patients not even in severe alcohol withdrawal since the onset is 2–3 hours and can only be given PO. The initial dose can be 50–100 mg PO.

Learners must also consider how further progression of alcohol withdrawal and pharmacological management can result in loss of airway protection and be prepared to intervene early on. While the mainstay of alcohol withdrawal treatment targets the underlying pathophysiology (the lack of GABA_A_ activation) via medications mentioned above, it is important for learners to also consider and treat other abnormalities such as hypokalemia, hypomagnesemia, hypophosphatemia, alcoholic ketoacidosis and other electrolyte abnormalities.^1^,^4^ Treatment of hypokalemia, hypomagnesemia and hypophosphatemia is individualized to each patient based on their electrolyte levels and ability to tolerate PO formulations. Based on laboratory evaluation, it is important to recognize alcoholic ketoacidosis and treat it with dextrose containing fluids. Lastly, patients with alcohol abuse commonly have vitamin deficiencies such as thiamine and folic acid. These deficiencies can be replaced with 100 mg IV thiamine and 1 mg folic acid IV if unable to tolerate PO.^1^,^4^ If a patient is able to tolerate PO, a patient may take oral multivitamins in addition to oral thiamine and folic acid supplementation.^1^,^4^ However, these patients are also at risk of Wernicke’s encephalopathy and Korsakoff’s psychosis. If there is concern for these underlying pathologies, patient should be treated with increased dose of thiamine 200–500 mg IV every 8 hours.^1^,^4^

**Other debriefing points:** For this case, it was important for learners to differentiate alcohol withdrawal from other causes of the altered mental status. A few different ways in which important information was conveyed included: asking the husband to stay in the room to discuss history, probing on social history, using props such as plastic ants to demonstrate to learners the tactile and visual hallucinations the patient was experiencing. The ants are a physical representation of what the patient is hallucinating. We placed the ants on left arm, right leg and abdomen. This gives the learner a peek into what the patient is experiencing. In reality, these ants would not physically be present; however, they are in the patient’s mind. If learners did not pick up these clues, during the debrief you could ask, “How did the husband impede or aid you in your diagnosis?”, “When asking about social history, were there any clues to dig deeper?” and “What did you think when you saw the plastic ants on the patient?”

Next, rapid administration of benzodiazepines, phenobarbital and other medications is important to the case. Learners, depending on their level of training, may have difficulty identifying the correct medications and correct dosage. During the debrief, you could ask, “What resources/clinical tools could you use to help you identify what medications and dosage you should use to treat the patient?” Lastly, an important step is identifying a critically ill patient unable to protect their airway. If learners did not identify this situation, you could ask, “When you see a patient unable to protect their airway, what goes through your head?”

## SIMULATION ASSESSMENT

*Alcohol Withdrawal with Delirium Tremens*

Learner: _________________________________________

***Assessment Timeline***

This timeline is to help observers assess their learners. It allows observer to make notes on when learners performed various tasks, which can help guide debriefing discussion.[Table t16-jetem-8-3-s1]

**Critical Actions:**

□ Obtain point of care (POC) glucose
□ Ask husband for collateral history of alcohol use
□ Identify alcohol withdrawal with delirium tremens
□ Initiate treatment with benzodiazepine and escalate to higher dosing of medication
□ Assess deep tendon reflexes for additional benzodiazepine dosing
□ Initiate phenobarbital treatment when alcohol withdrawal is refractory to benzodiazepine administration
□ Recognize worsening mental status and establish airway
□ Admit patient to ICU

**Summative and formative comments:**

**Milestones assessment:** [Table t17-jetem-8-3-s1]
