# Supplementary material for: Alcohol Withdrawal with Delirium Tremens
Source: J Educ Teach Emerg Med. 2023 Jul 31;8(3):S1–S33. doi: 10.21980/J8S35N (PMC10414982; doi:10.21980/J8S35N)

## Slide 1
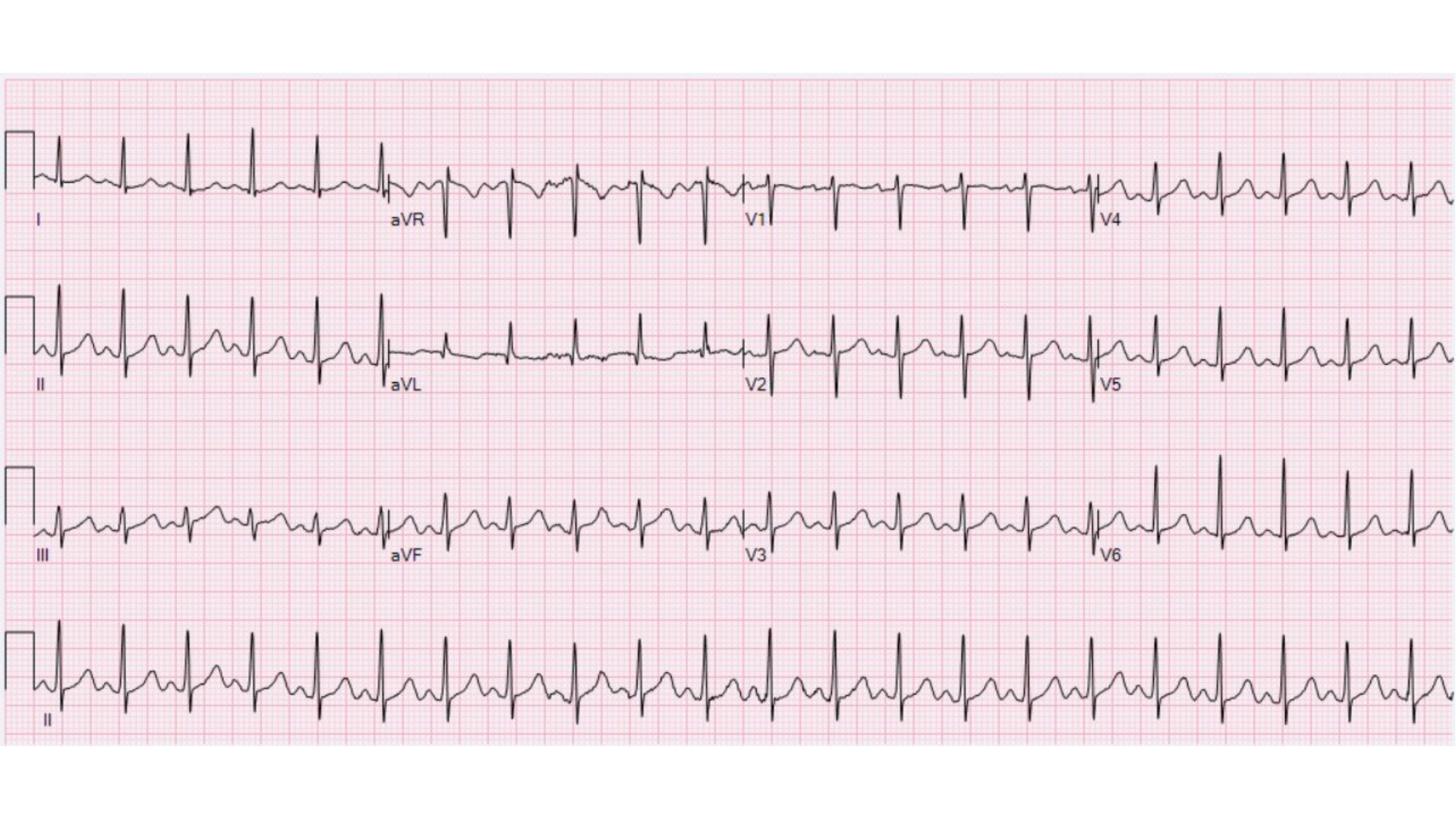

## Slide 2
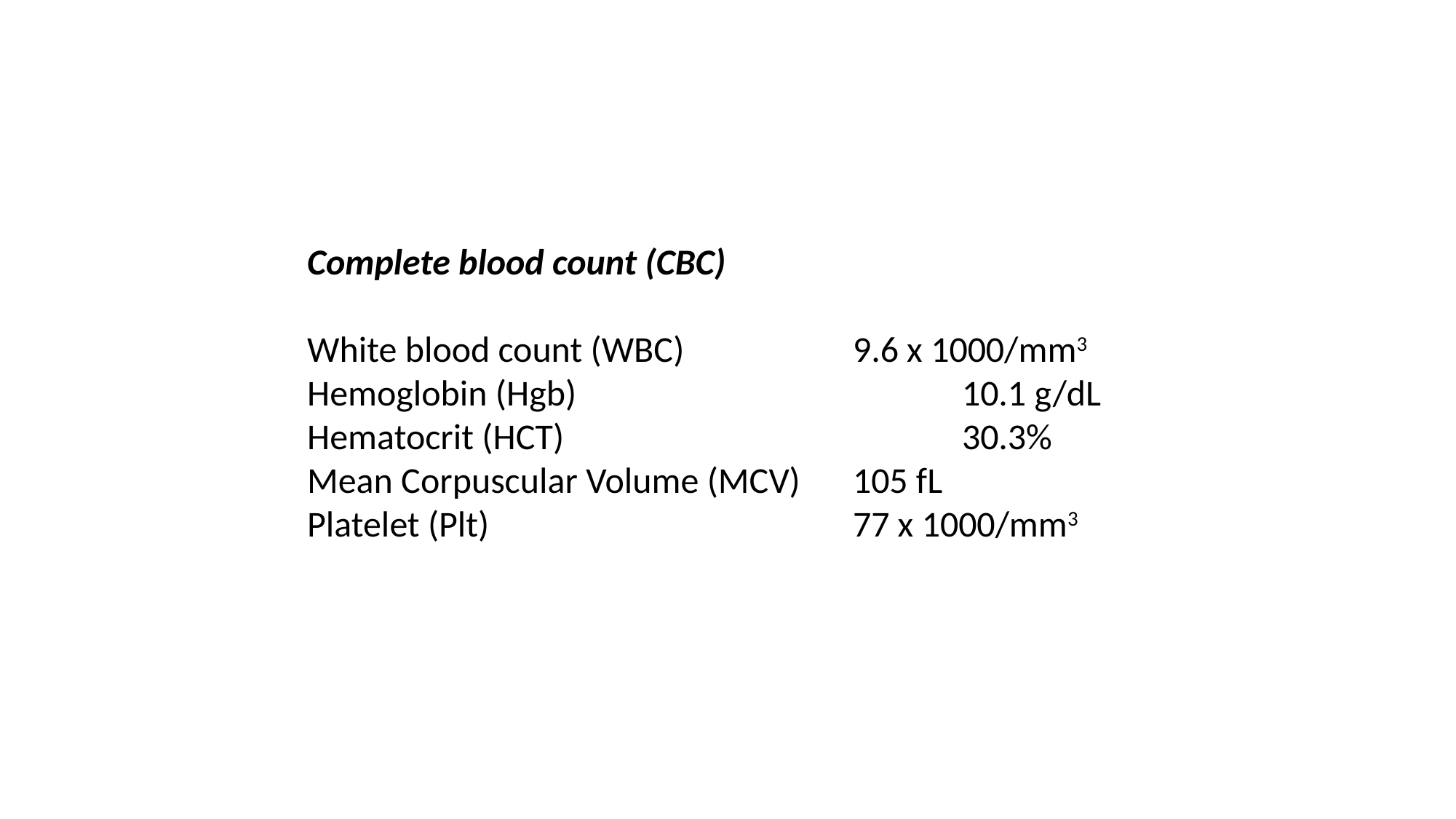

Complete blood count (CBC)
White blood count (WBC)		9.6 x 1000/mm3
Hemoglobin (Hgb)				10.1 g/dL
Hematocrit (HCT)				30.3%
Mean Corpuscular Volume (MCV)	105 fL
Platelet (Plt)				77 x 1000/mm3

## Slide 3
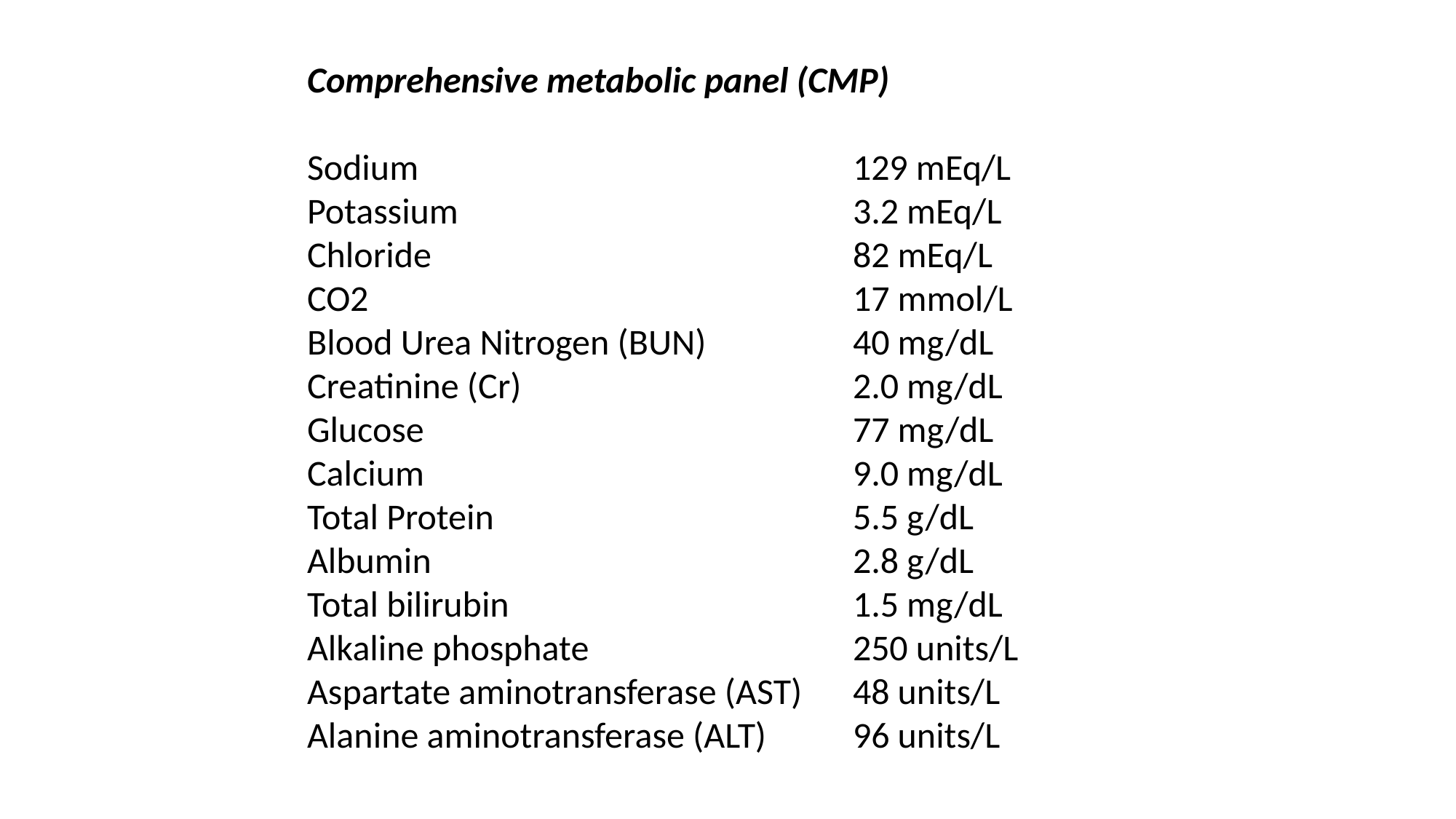

Comprehensive metabolic panel (CMP)
Sodium					129 mEq/L
Potassium					3.2 mEq/L
Chloride					82 mEq/L
CO2					17 mmol/L
Blood Urea Nitrogen (BUN)		40 mg/dL
Creatinine (Cr)				2.0 mg/dL
Glucose					77 mg/dL
Calcium					9.0 mg/dL
Total Protein				5.5 g/dL
Albumin					2.8 g/dL
Total bilirubin				1.5 mg/dL
Alkaline phosphate			250 units/L
Aspartate aminotransferase (AST)	48 units/L
Alanine aminotransferase (ALT)	96 units/L

## Slide 4
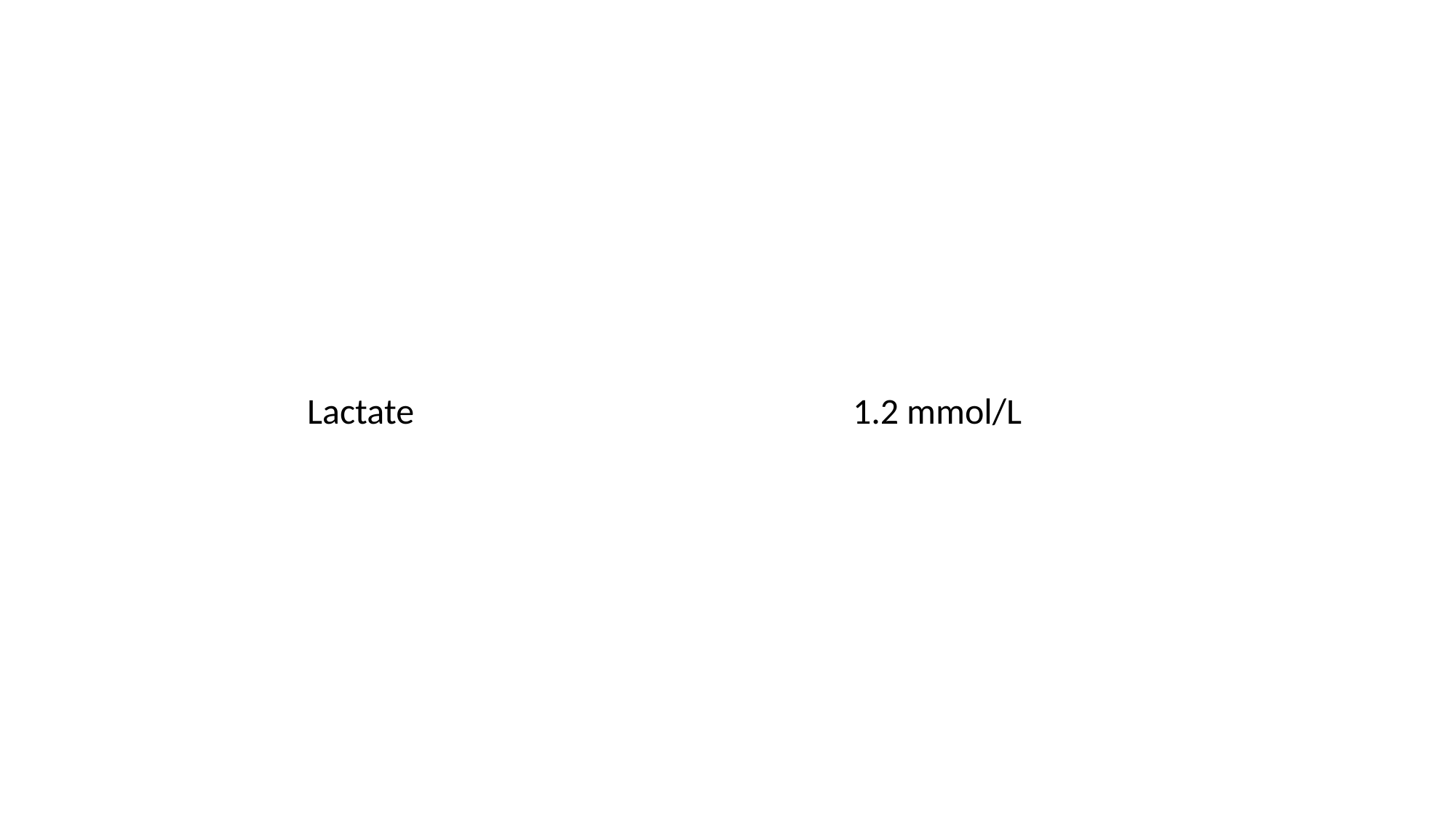

Lactate					1.2 mmol/L

## Slide 5
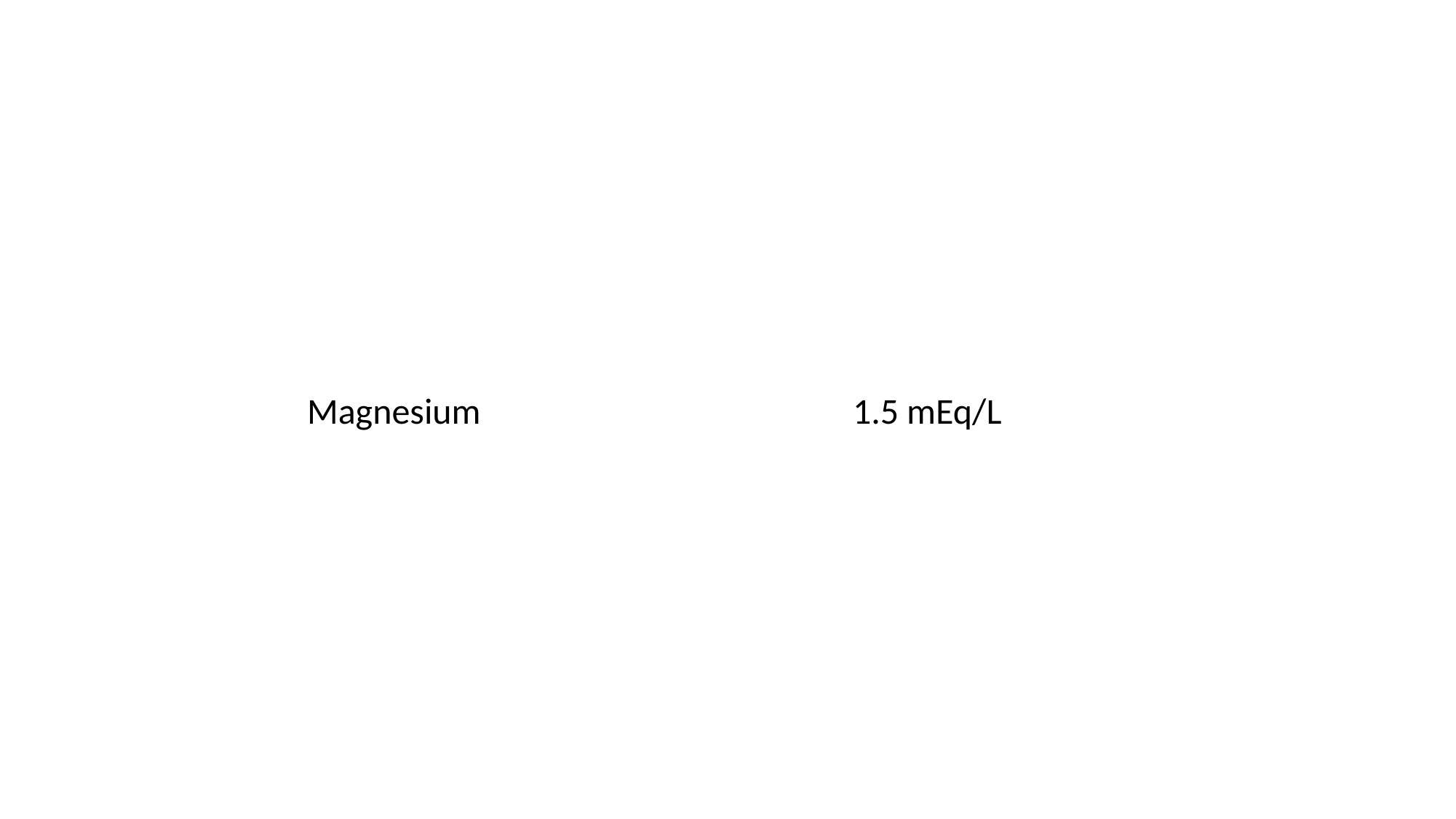

Magnesium					1.5 mEq/L

## Slide 6
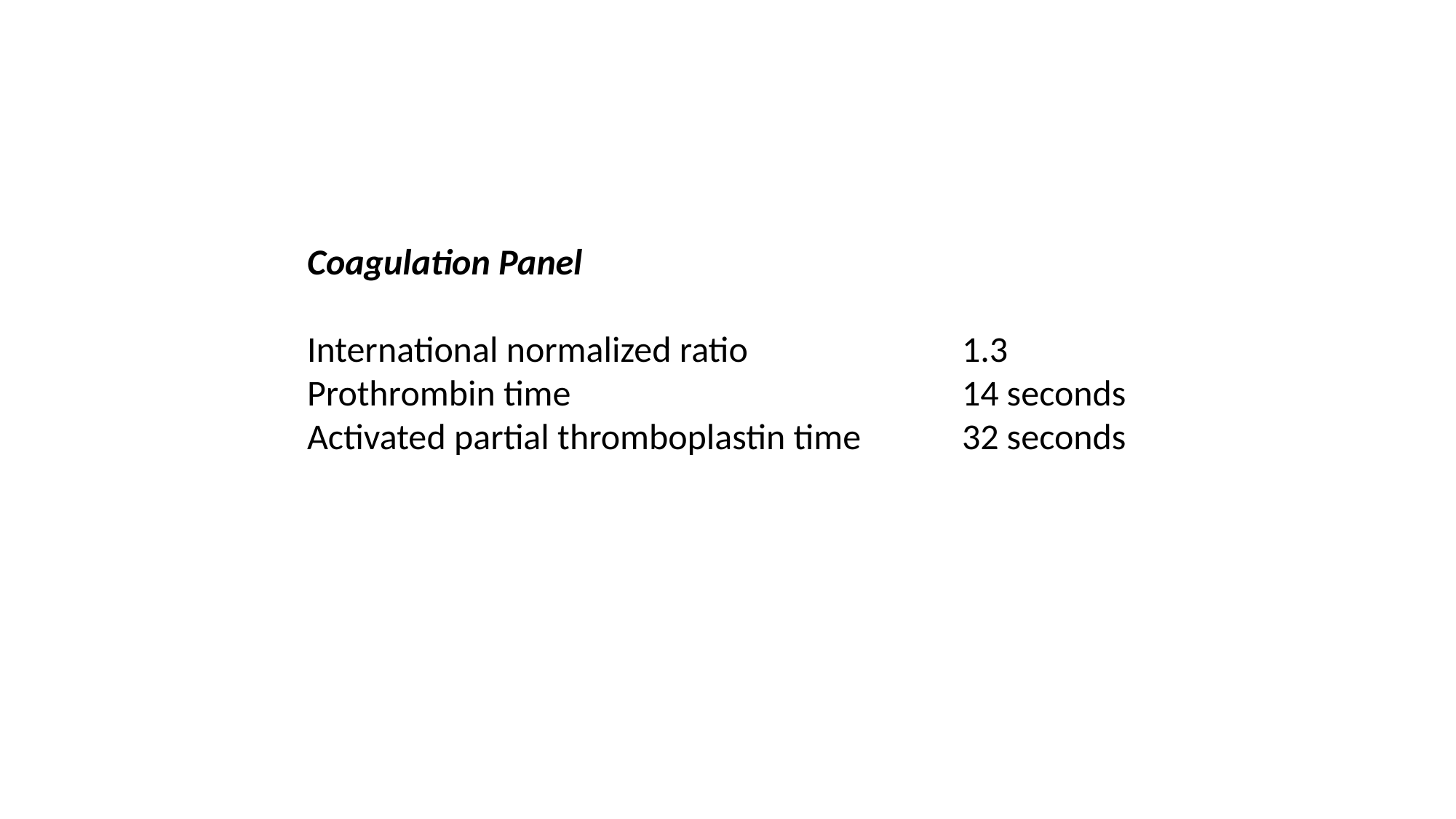

Coagulation Panel
International normalized ratio 		1.3
Prothrombin time 				14 seconds
Activated partial thromboplastin time 	32 seconds

## Slide 7
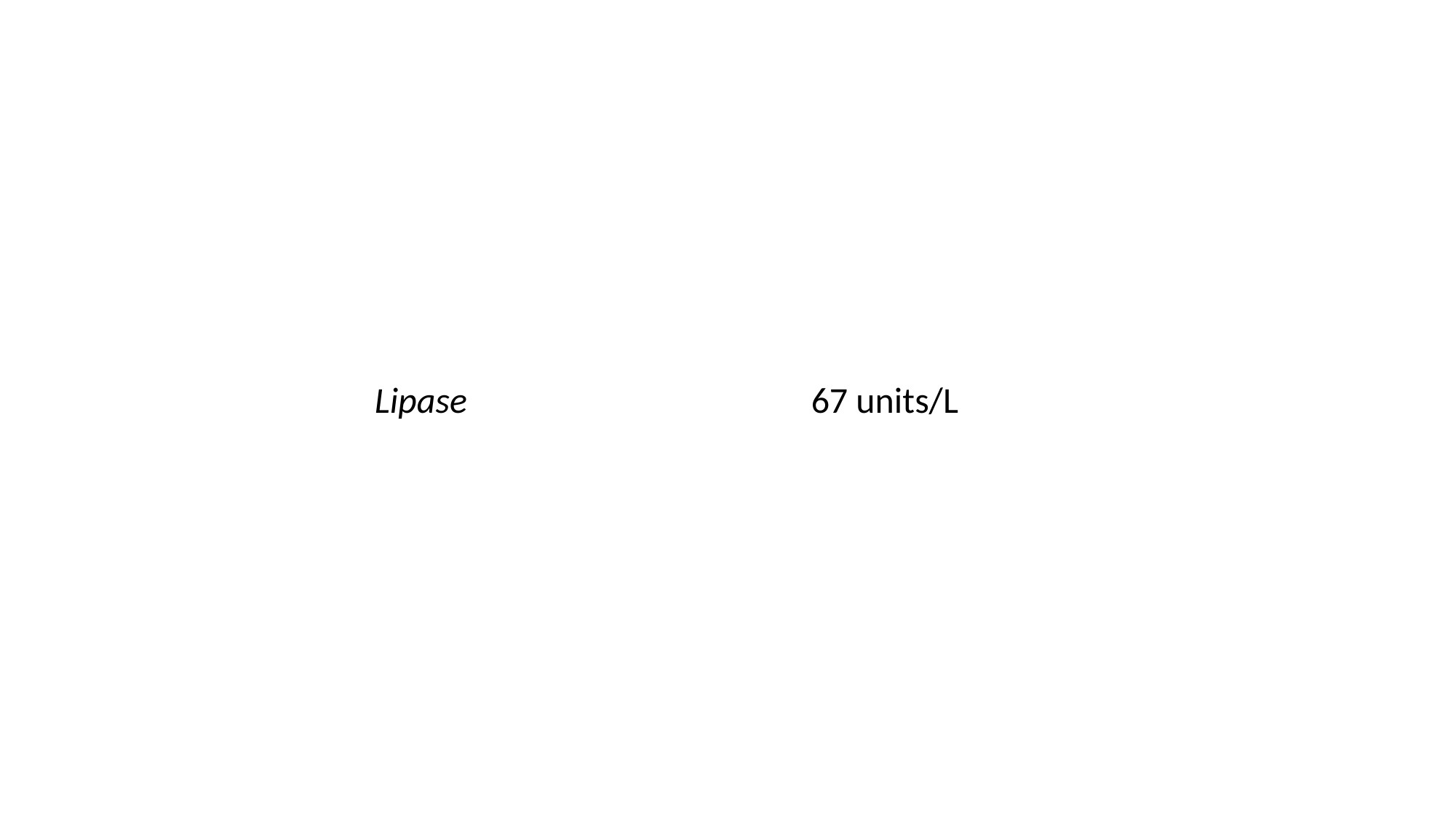

Lipase				67 units/L

## Slide 8
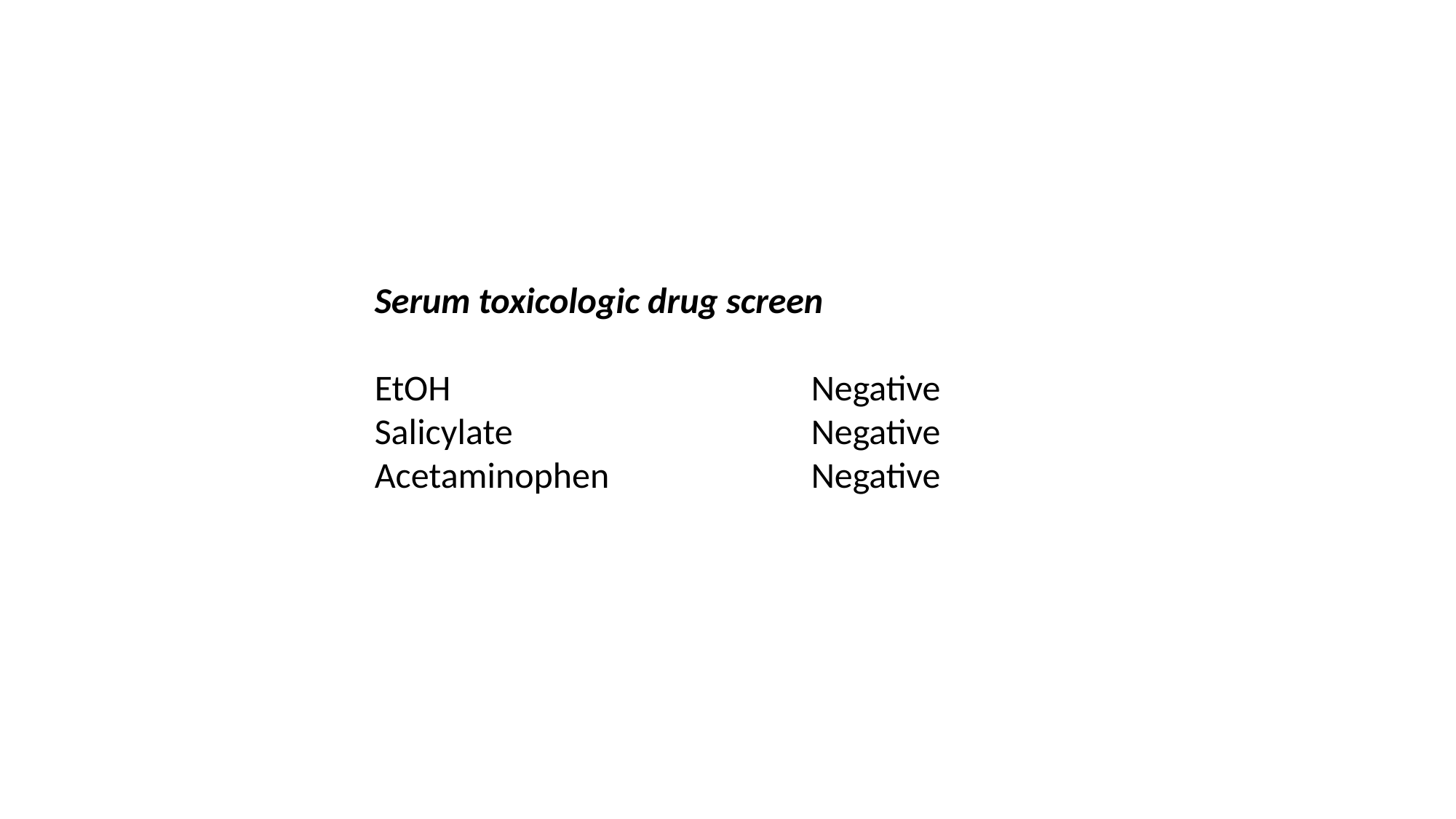

Serum toxicologic drug screen
EtOH				Negative
Salicylate			Negative
Acetaminophen 		Negative

## Slide 9
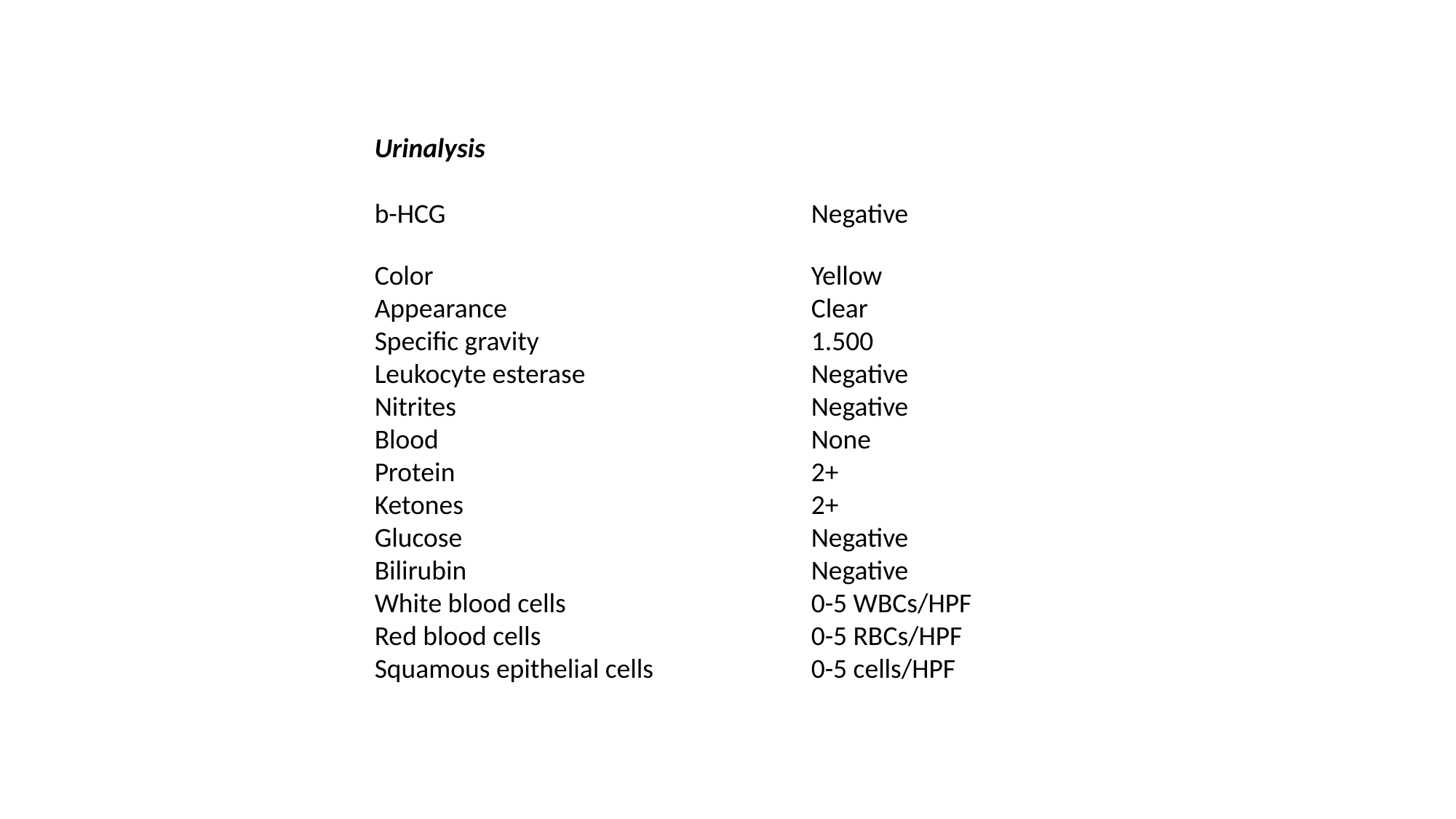

Urinalysis
b-HCG				Negative
Color				Yellow
Appearance			Clear
Specific gravity			1.500
Leukocyte esterase			Negative
Nitrites				Negative
Blood				None
Protein				2+
Ketones				2+
Glucose				Negative
Bilirubin				Negative
White blood cells			0-5 WBCs/HPF
Red blood cells			0-5 RBCs/HPF
Squamous epithelial cells		0-5 cells/HPF

## Slide 10
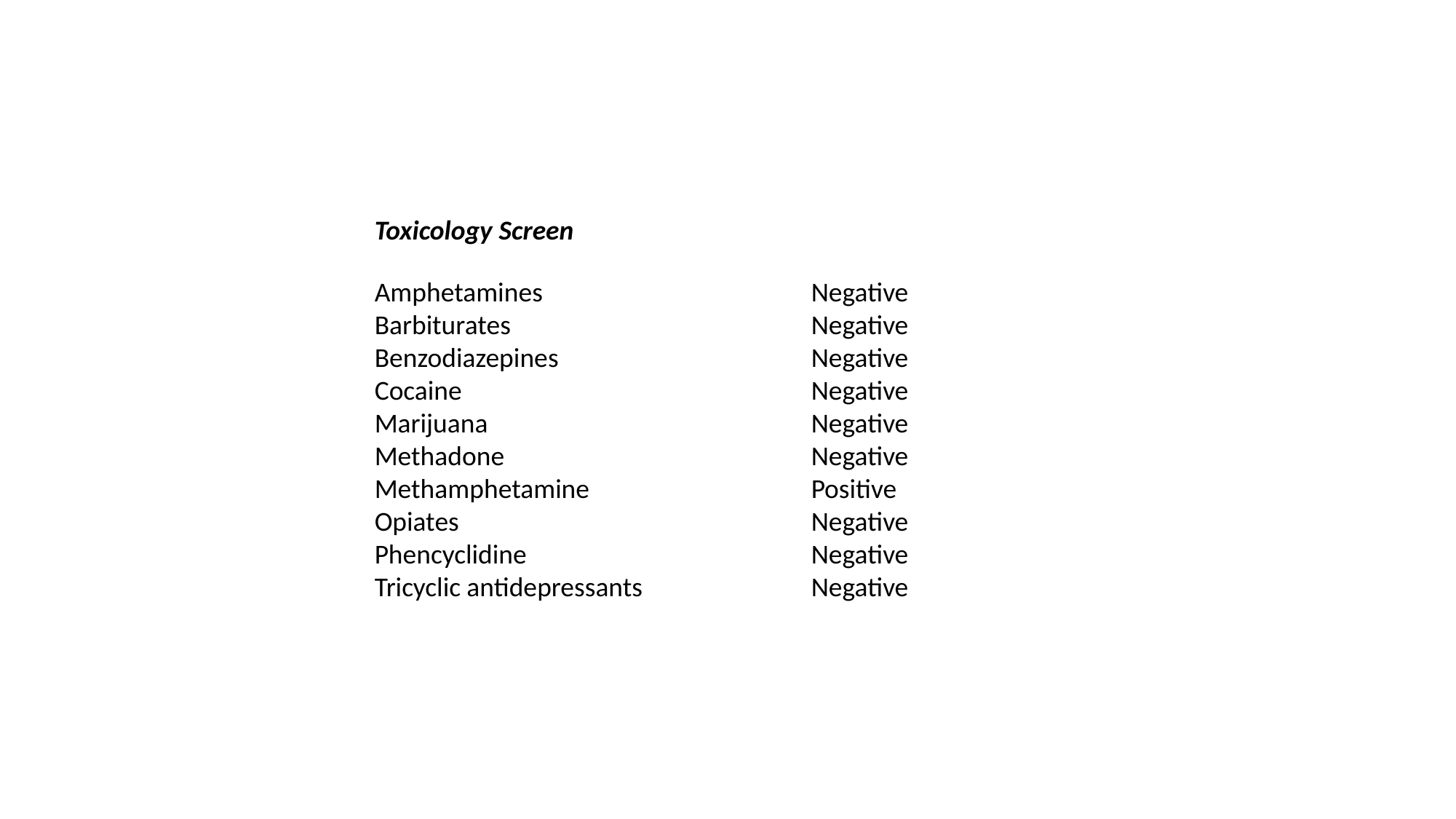

Toxicology Screen
Amphetamines			Negative
Barbiturates			Negative
Benzodiazepines			Negative
Cocaine				Negative
Marijuana			Negative
Methadone			Negative
Methamphetamine			Positive
Opiates				Negative
Phencyclidine			Negative
Tricyclic antidepressants		Negative

## Slide 11
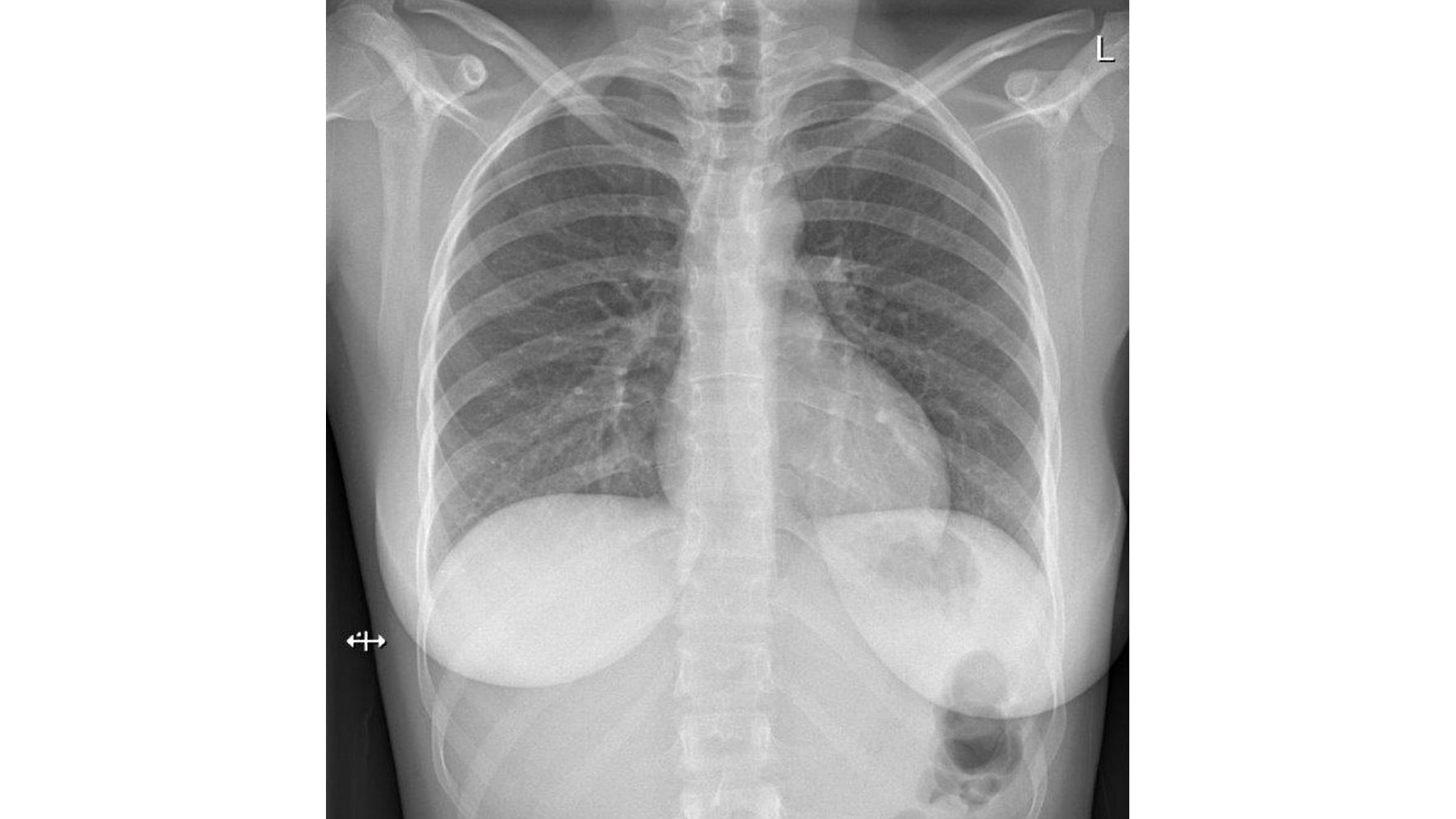

## Slide 12
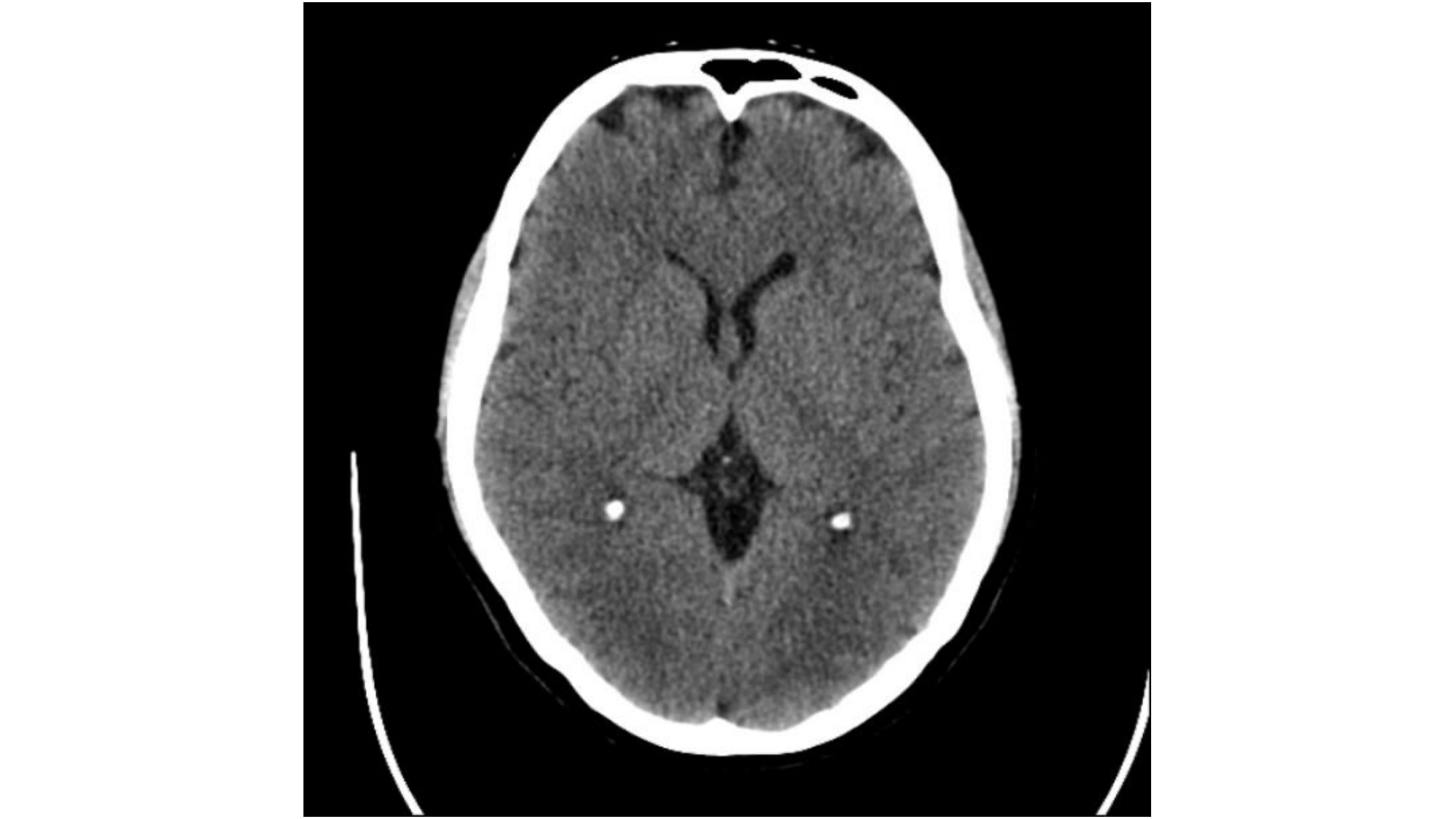

Supplement: Supplementary file 1 [file JETem-8-3-S1-supp1.pptx]
